# Application of a Fuzzy Logic Based Methodology to Validate the Hydrochemical Characterization and Determining Seasonal Influence of a Watershed Affected by Acid Mine Drainage

**DOI:** 10.3390/ijerph18094693

**Published:** 2021-04-28

**Authors:** Jose M. Davila, Aguasanta M. Sarmiento, Javier Aroba, Juan C. Fortes, Jose A. Grande, Maria Santisteban, Francisco Cordoba, Mercedes Leiva, Ana T. Luís

**Affiliations:** 1 Department of Water, Mining and Environment, Scientific and Technological Center of Huelva, University of Huelva, 21007 Huelva, Spain; amsarmiento@uhu.es (A.M.S.); jcfortes@dimme.uhu.es (J.C.F.); grangil@dimme.uhu.es (J.A.G.); maria.santisteban@dimme.uhu.es (M.S.); anatluis@ua.pt (A.T.L.); 2 Sustainable Mining Engineering Research Group, Department of Mining, Mechanic, Energetic and Construction Engineering, Higher Technical School of Engineering, University of Huelva, 21007 Huelva, Spain; 3 Department of Information Technologies, Higher Technical School of Engineering, University of Huelva, 21071 Huelva, Spain; aroba@dti.uhu.es; 4 Department of Integrated Sciences, Faculty of Experimental Sciences, University of Huelva, 21007 Huelva, Spain; cordobagarcia@gmail.com (F.C.); mercedeslys@gmail.com (M.L.); 5 Department of Mining, Mechanic, Energetic and Construction Engineering, Higher Technical School of Engineering, University of Huelva, 21007 Huelva, Spain; 6 GeoBioTec Research Unit, Department of Geosciences, University of Aveiro, Campus de Santiago, 3810-193 Aveiro, Portugal

**Keywords:** Odiel River Basin, seasonal influence, Iberian Pyrite Belt, metal concentrations, acidity, polluted water

## Abstract

The Odiel River Basin, located in the Iberian Pyrite Belt (IPB), is heavily affected by acid mine drainage (AMD), which occurs when pyritic minerals from sulfide mining areas are exposed to atmospheric, hydrological or biological weathering. This paper presents a hydrochemical characterization of parameters in the Odiel River Basin by means of Fuzzy Logic and data mining methodologies to determine the seasonal influence of AMD in polluted waters that have not been used before for a basin in this environmental area. This technique was proven to be effective, providing results that could not be achieved by using classic statistics, because it allows us to characterize the different parameters separately and also their relationships in waters affected by AMD in a qualitative manner based on the antecedents and according to the conditions (rules) imposed by the consequents (in this case, the Fe(II) and accumulated rainfall over 30 days). Thus, it was possible to confirm that hydrochemistry is greatly affected by seasonal changes, with a higher pH in the wet season (up to 8.59) compared to 2.12, the minimum pH value reached in the dry season. Accordingly, higher concentrations of most of the metals were observed in the dry season (e.g., up to 4000 mg/L of Fe (II)), with the exception of the values found after the first rains that occur in the early fall. With the use of the Fuzzy Logic technique, it was observed that, during the wet season, lixiviates with a higher Fe content have higher metal concentrations, and in the dry season, the behavior is the opposite.

## 1. Introduction

Acid mine drainage (AMD) is one of the main environmental problems caused by the oxidation of sulfide-rich deposits after their extraction, when they are exposed to oxygen, water and biological weathering. In many cases, mining waste can continue to affect the environment negatively for centuries after mine closure, contaminating the surrounding soil, surface water and groundwater [1,2,3]. Acid mine drainage is related to the oxidation of pyrite and other poly-metallic sulfides, resulting in an acid lixiviate with high concentrations of metals and metalloids.

AMD-processes are due to the extraction of sulfide ores, occurring when pyrite is combined with oxygen in rivers or groundwater. In this evolution, there is a decrease in pH (below 3) and an increase in metal and sulfate concentrations [4,5]. The main reactions that occur in AMD environment were established by References [4,6], with these being the oxidation of pyrite (Equations (1) and (2)), oxidation of ferrous (Equation (3)) and precipitation of ferric (Equation (4)):

FeS_2_ + 7/2O_2_ + H_2_O → Fe^2+^ + 2SO_4_^2−^ + 2H^+^ (1)

Fe^2+^ + 1/4 O_2_ + H^+^→ Fe^3+^ + 1/2H_2_O (2)

FeS_2(s)_ + 1/4Fe^3+^ + 8H_2_O → 15Fe^2+^ + 2SO_4_^=^ + 16H^+^ (3)

Fe^3+^ + 3H_2_O → Fe(OH)_3(s)_ +3H^+^ (4)

**Figure 1:**
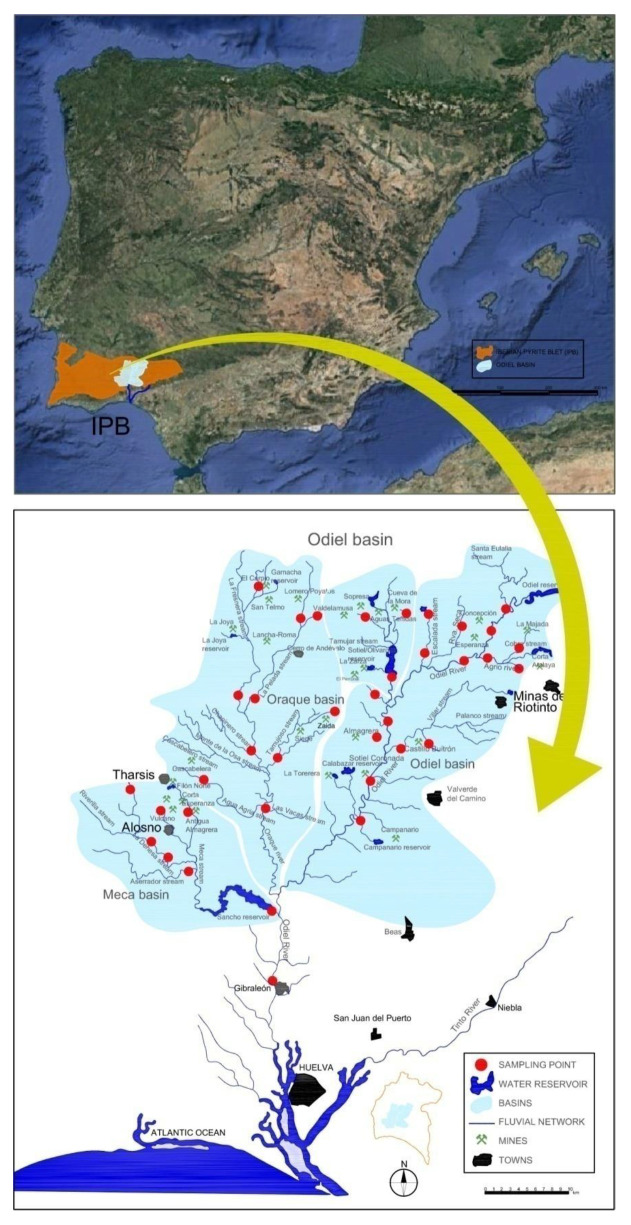
Location setting. It can be seen the Iberian Pyrite Belt (**up**) and the Odiel Basin (**down**).

In the southwest of the Iberian Peninsula, thousands of years of mining in the Iberian Pyrite Belt [7,8] have produced enormous amounts of metal sulfide waste that constitutes a serious threat to the environment. The Odiel River (Figure 1) is the main river in the Huelva province (or district), with 37% of its drainage network affected by AMD, which increases to almost 100% during dry seasons (in this area, it runs from March to September) [9].

However, this problem is not only localized in the IPB, being well-known worldwide, with a lot of research works done, addressing the problem of contamination by AMD in rivers and also in groundwater systems [10,11,12]. Some ideas for its remediation have been highlighted in scientific works [13,14,15]; although, generally, the solutions found until now have been difficult to apply, due to high costs and high AMD extension areas.

A large number of studies have aimed to evaluate the processes that control the hydrochemistry of the contaminating elements in both the fluvial course [16,17,18,19] and the estuarine mixture once the Odiel River flows into the Gulf of Cadiz [20,21,22,23,24].

The Odiel River is the largest hydrographic network in the Huelva province, with 1149 km of rivers and streams, and together with the Tinto River, it flows into the Atlantic Ocean, forming an estuary that constitutes an important biosphere reserve. However, the Odiel River is highly polluted in much of its route, which is confirmed by the loads transported by the river to the ocean that have been set at 820 t/day of sulfates and 45 t/day of metals (mainly Fe, Zn, Mn, Cu, Pd and Cd) [25].

Two important reservoirs are located in the studied basin, the Olivargas and the Sancho, both affected by AMD, and the construction of the Alcolea Reservoir has begun, which under current conditions, will be of acidic water. The level of contamination is also confirmed by the low pH values (1.66) and the high concentrations (e.g., of up to 2157 mg/L Al, 117 mg/L As and 23889 mg/L total Fe) measured by the authors [26] in the area of mining waste located in the estuary.

The authors of Reference [9] carried out an exhaustive study on the whole basin, focusing on the level of pollution and hydrochemical characteristics. A significant amount of data was obtained throughout a complete hydrological year, from which relevant conclusions were achieved. However, despite the large amount of data obtained, no investigations focused on the relationships between the different physical–chemical parameters analyzed. This target would not be easy to be achieved with classical statistics, whereas the Fuzzy Logic technique is easier to perform and interpret.

Although classical statistics is a useful methodology for modeling this type of system [22,27,28] by using tools such as Cluster Analysis, Factorial Analysis and Principal Component Analysis, Fuzzy Logic goes one step forward, allowing more information to be extracted from the same database. The reason for this is that classical statistics works by means of a binary system of “ON-OFF”, “YES-NO” or “White-Black”. On the contrary, Fuzzy Logic allows for discriminating a whole range of intermediate values; for example, between white and black, a multitude of grays could be defined. It is for this reason that this technique allows for the very good qualitative modeling of complex systems with several variables, having also been used before by this working team in similar systems for the establishment of cause–effect relationships in mining-affected watercourses [5,29,30].

This paper presents a new approach for characterizing the seasonal influence on the level of pollution and hydrochemical characteristics in an AMD-affected river (the Odiel River). The proposed methodology is based on the data-mining computer tool PreFuRGe [31] that has proved to be suitable for modeling the qualitative behavior of complex systems [5,29] and provides greater consistency than classical statistics [30].

In the context of this research, the Fuzzy Logic has been used to address some problems, such as changes in the spatial variability of hydraulic parameters [32], identification of quality indexes of groundwater [33], distribution of hydrochemical facies [34] or prediction of a rock engineering classification system [35].

This paper proposes (and this constitutes its aim) the application of a Fuzzy Logic and data-mining-based methodology to characterize the influence of seasonal changes on the level of pollution and hydrochemical characteristics in the Odiel River (affected by AMD). The obtained results will be compared with previous works in the same area, and the veracity and importance of the qualitative information generated by PreFuRGe tool will be checked.

## 2. Site Description

The Odiel River Basin flows mostly through the Iberian Pyrite Belt (IPB—Figure 1). The IPB extends from Seville, passing through the province of Huelva from east to west, and ends in Portugal (Alentejo). It is considered the largest massive polymetallic sulfide deposit in the world, being more than 200 km in length and 40 km wide, with an estimated sulfide ore reserve of 1700 Mt [36]. More than 80 massive polymetallic sulfides deposits are found in the IPB, with pyrite (FeS_2_) being the main mineral, together with smaller quantities of sphalerite (ZnS), galena (PbS), chalcopyrite (CuFeS_2_), arsenopyrite (FeAsS) and other sulfide minerals with low contents of Cd, Sn, Ag, Au, Co, Hg, etc. Figure 1 shows the 28 main mines distributed in the Odiel Basin, such as Riotinto, Tharsis, Confesionarios, Sotiel, etc.

From a geological point of view, the sequence of IPB is classified as Culm, in which sandstones, conglomerates and shales predominate; Phyllite–Quartzite group, formed by sandstones and shales of great thickness; and, finally, the Volcano–Sedimentary Complex, in which a succession of mafic–felsic volcanic materials interstratified with shales can be observed. The Culm, Phyllite Quarzite and Volcano–Sedimentary groups are of Upper Palezoic age, the latter of which is hosting the polymetallic sulfide ore deposits.

The Odiel Basin is located in the SW of the Iberian Peninsula (Figure 1) and is the largest hydrographic basin in the province of Huelva, with an area of about 2300 km^2^. The Odiel River originates at the Sierra de Aracena and, together with the Tinto River, flows into a coastal wetland known as Ria de Huelva, which forms part of a very important Natural Reserve (Figure 1). The Odiel River basin is divided into three main sub-basins called Odiel, Oraque and Meca (see Figure 1). The Odiel River flow has been estimated at around 500 hm^3^/year [37], although the variations in this average are due to the Mediterranean climate, which includes long periods of drought and intense rainfall. The annual average rainfall value in the basin is 812 mm, 50% of which occurs between October and February (wet season) [38].

## 3. Materials and Methods

### 3.1. Sampling Points

In the 2003/2004 hydrological year, 37 surface-water sites were collected from the Odiel Basin; 111 samples and 121 samples were sampled in the wet and dry seasons, respectively; each sampling session was carried out in a period of 35 and 40 days, respectively. It must be taken into account that the number of samples was reduced in the summer period, since some streams were dried. All the sampled points belonged to streams affected by acid lixiviates from different mines located in Figure 1. Precipitation data were obtained from three rainfall stations located in different parts of the Odiel basin. The accumulated rainfall for 30 days prior to each sampling was used to study the seasonal variation in pollution.

Water samples were filtered directly in the field at 0.22 µm (Millipore filters installed in syringes). Samples for cations and metal analysis were acidified in the field at pH < 2 with Suprapur HNO_3_ (2%). They were then stored in the dark, at 4°C, in polyethylene bottles, until analysis. The samples for the determination of Fe(II) were filtered to 0.1 µm and buffered at pH = 4.5 with an ammonium acetate/acetic buffer, following the methodology established by Reference [39].

### 3.2. Analytical Procedure

The field parameters (temperature, pH and electrical conductivity) were measured by using a portable MX 300 m (Mettler Toledo). Dissolved oxygen was measured with a Hanna meter. Finally, the redox potential was determined by using a Pt electrode (Hanna), that was previously calibrated by using Hanna standard solutions (pH 4.01 and 7.01) for the pH and Hanna standard solutions (240 and 470 mV) for Eh.

The concentrations of dissolved Al, As, Ca, Cd, Co, Cr, Cu, Fe, K, Mg, Mn, Na, Ni, Pb, Sb, Se, Si, Sn and Zn were determined by using Inductive Coupling Plasma Atomic Emission Spectrometry (ICP–AES Yobin-Ybon Ultima2). The analysis was carried out at the Central Research Services of the University of Huelva. For calibration, multi-element standard solutions prepared from unique certified standards supplied by SCP SCIENCE were used. It was performed at the beginning and end of each analytical series. The SRM-1640 NIST certified freshwater-type reference material and the IRMM-N3 inter-laboratory standard, a wastewater test material from the European Commission’s Institute for Reference Materials and Measurements, were also analyzed. The detection limits were calculated as the average and the standard deviations of ten blanks. The detection limits for larger cations were 200 µg/L for Al, Fe, Mn, Mg, Na, K and Si; and 500 µg/L for Ca. For trace elements, they were 50 µg/L for Zn, 5 µg/L for Cu, 2 µg/L for As and 1 µg/L for the rest of the elements. The precision was better than 10% in all analyzed elements.

Fe(II) was determined using colorimetry at 510 nm with a SHIMADZU UV mini-1240 spectrophotometer, and after complexing a 0.5% (*w*/*w*) 1,10-phenanthrolinium chloride solution was added to the filtered sample [39]. The detection limit was 0.3 mg/L, and the precision was better than 5%.

### 3.3. Data Mining and Fuzzy Logic

Computer tools based on Data Mining enable the extraction of meaningful (and sometimes unknown) information, based on the used stored data. With these tools, the results obtained can go further than the information obtained through classical statistical techniques [40,41]. It is very useful for the experts that have to interpret the results, to be able to analyze them qualitatively by using a natural language. By using Fuzzy Logic–based methodologies, the results can be interpreted in the same way as a they would be by humans [42,43,44], since the graphs generated by the software can be interpreted visually (this is explained in more detail in Section 3.3.2.)

Fuzzy Logic [45] operates by using rules of reasoning quite similar to the imprecise, intuitive and human way of thinking, allowing for characterizing variables without specifying a precise value by using a membership grade, something that is not possible to get with classical logic (binary), in which an element belongs (or not) to a set. Thus, the classical statistics would work correctly in those cases in which the values are very low or very high. A problem may occur when dealing with intermediate values or with a wide range of values. In these cases, the Fuzzy Logic technique should be applied, allowing us to define exactly the certainty degree with respect to a given expression, e.g., “the pH value is very low”.

The Fuzzy Logic technique has been used successfully in analogous situations in several countries [46,47] with satisfactory results.

#### 3.3.1. Fuzzy Clustering

Clustering algorithms [48] classify a dataset into groups (clusters) in which their elements are more similar to other elements in the rest of the clusters. While in classical clustering algorithms, each element is assigned to one cluster (meaning that it membership grade can only take the value 0 or 1), in fuzzy clustering algorithms [49] an element may belong to more than one cluster, because the membership grade of each element can take real values between 0 and 1 (partial membership grade).

One of the most used fuzzy clustering algorithms is the Fuzzy C-Means (FCM) algorithm [49,50,51].

#### 3.3.2. PreFuRGe Methodology (Predictive Fuzzy Rules Generator)

**Figure 2:**
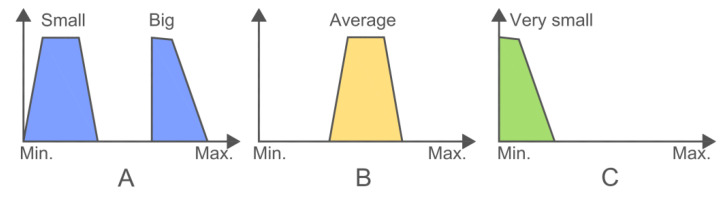
Example of If–Then fuzzy rule. The antecedent parameters are (**A**) and (**B**), and the consequent is (**C**).

The first step before the data processing is the selection of the objective parameters whose behavior is of interest for experts (e.g., DO, pH, Fe, Al, Pb, etc.). These goal parameters will be the consequents in the obtained fuzzy rules, and, therefore, the rest of the remaining parameters will be the antecedents (Figure 2).

PreFuRGe analyzes the provided dataset, so that the selected goal parameters (consequent), are organized in an optimum number of fuzzy clusters [31,50]. Then, each fuzzy cluster is projected onto the antecedent space [52], to determine the membership grade of the antecedents to the fuzzy clusters.

If the minimum distances between data (or points) are taken into account and they are compared with a standard value, the variables can be grouped by using the Fuzzy C-Means algorithm [53] (Equation (5)) in the four clusters. Finally, once the previous described values are determined, the graphical fuzzy rules are generated (Figure 2).

(5) $$J\left(X;U,V\right)=\sum_{i=1}^{c}\sum_{k=1}^{n}{\left(\mu_{\mathrm{ik}}\right)}^{m}\|x_{k}-v_{i}{\|}_{A}^{2}$$

where U is a fuzzy partition matrix of X, V is a vector that is used to determine the cluster centers, m is a coefficient that measures the degree of concordance of the resulting groups, μ_ik_ can take the values 0 ≤ μ_ik_≤ 1, and $\|x_{k}-v_{i}{\|}_{A}^{2}$ is (x_k_− v_i_)T A(x_k_− v_i_) and is used for measuring distances.

PreFuRGe provides a graphical output that enables an easy interpretation of the fuzzy rules in natural language. The main characteristics of the provided fuzzy rules are as follows:

- The fuzzy set assigned to each parameter is represented by a trapezium,
- The parameters values are represented on the *x*-axis of each fuzzy set,
- The parameters membership grade to a cluster is represented on the *y*-axis.

In the fuzzy rule represented in Figure 2, the antecedent parameters are A and B, and the consequent is C. This fuzzy rule would be interpreted as follows: IF A is small OR big AND B is average, THEN C is very small. This type of relationship between antecedents and consequents allows us to relate the variables even when the coincidence is not complete. Thus, this technique is especially effective when many parameters and the relationship among them are evaluated.

## 4. Results and Discussion

### 4.1. Hydrochemistry of the Odiel River Basin

Table 1 shows the universe of the analyzed parameters for one the list of 37 sampling points. It can be observed that the physicochemical parameters’ range is highly variable, with annual ranges from 2.1 to 8.8 for pH; 0.1 to 18.5 mS/cm for electrical conductivity; and element concentrations of up to 2045 mg/L Al, 7.5 mg/L As, 4282 mg/L total Fe and 36,397 mg/L sulfates, among others. These high concentrations have a very negative effect on the health and to the biota of this area, since there are several reservoirs in the basin, and these waters reach the estuary located near the river mouth.

Hydrochemical AMD processes in the Odiel River Basin show different patterns depending on factors such as seasonal variations and contamination levels [9]. Regarding the contamination levels, the slightly affected streams did not undergo great changes in their chemical composition, although there were clear differences between the wet season and the dry season. On the other hand, the highly affected streams were strongly influenced by the season, especially by rainfall events, because rain lixiviates the minerals that are dragged to the river. However, in both cases the level of contamination increased as rainfall decreased (because the dilution processes are reduced); the metal/Fe ratio did not undergo the same evolution. The metal/Fe ratio increased from the wet to the dry season in the slightly affected samples, while it decreased in the strongly affected samples [9]. This different behavior is due to the lower mobility of Fe in the slightly affected streams than in the streams highly affected by AMD. While in the strongly affected streams, the precipitation of Fe decreases as a consequence of the sharp drop in pH, in the slightly affected streams, the decrease in pH is not so strong, so that the Fe precipitates as a consequence of evaporation.

During the dry season, the water contributions to the rivers are almost entirely due to lixiviates from mining operations, so it is usually in the months of July and August when the lowest pH values are recorded.

To the best of our knowledge, the approach with the Fuzzy Logic technique was not applied in other basins but in the IPB. Our results are expected to be relevant, both due to their extension (a complete basin) and the great accuracy the Fuzzy Logic technique has.

In Reference [54], the authors limited themselves to studying the Sanae stream, finding that the concentrations of dissolved metals and sulfates were higher in autumn, coinciding with dry season and obviously when the lowest pH values were determined (in the autumn months). Meanwhile, in the research developed in Reference [55], the authors began to study the seasonal influence of the monsoons in India.

An analysis similar to ours was carried out in the Puna region (Argentina) [56] with a climatology similar to the one of the studied area (with the dry season coinciding with the austral winter). However, this research was conducted based on data obtained from points close to the mining exploitation (e.g., tailings, dams and not from the entire basin), determining an increase in pH at the end of the wet season together with a decrease in metal concentrations.

It was possible to study other antecedents that address the seasonal influence in AMD environments, but in this case [57,58,59], in reservoirs or lakes. However, these situations are not comparable to the one studied here, since a river is a transport medium, while a lake is a receptor medium in which the kinetics and hydrochemistry are very different [60,61,62], and the seasonal influence is smaller once the variations in the water volume due to rain are much less than that in rivers. In this scenario, the climate was Mediterranean subtropical, and due to this, rainfall is sometimes torrential, causing very significant variations in the rivers due to dissolution, while in the reservoirs, the volume stored in them exerts a buffering effect on the seasonal variations. However, the hydrochemistry of the most lakes varies remarkably with depth, depending mainly on its deepness [60,61].

With respect to seasonal variations, it was observed that, in the dry season, the maximum and average concentrations were higher for all of the analyzed elements, except for arsenic and lead, which were higher in the rainy season. In the dry season, the average pH of the basin was 3.8 (with minima up to 2.1), and the average concentrations of metals were 186 mg/L of Al, 1.6 mg/L Co, 317 mg/L Fe, 70 mg/L Zn, 178 μg/L Pb and 162 μg/L As, etc. Meanwhile, in the wet season the average pH was 4.1 (with minima up to 2.5), and average metal concentrations were mainly 82 mg/L Al, 0.8 mg/L Co, 133 mg/L Fe, 31 mg/L Zn, 275 μg/L Pb and 245 μg/L As.

Increases in Pb in the wet months were also observed in other rivers from the IPB [62]. Lead has a strong affinity to co-precipitate and/or adsorb onto Fe oxy-hydroxy-sulfates. The increase observed could be due to initial oxy-hydroxy-sulfates that retain Pb, followed by a subsequent release through desorption or transformation processes of these mineral phases [63]. The lower concentrations of As observed in summer (period included in the dry season) may be due to the speciation undergone in this element. Arsenic is strongly adsorbed onto Fe(III)-oxyhydroxides precipitates, especially As(V). The proportion of As(V) increased during the dry season, due to oxidation processes, which are more favorable in the summer season [64]. Arsenite oxidation is slow, especially under acidic conditions, but may be catalyzed by the activity of bacteria such as *Thiomonas*sp. [65], thus increasing its activity in summer. Furthermore, arsenic species are also strongly influenced byphoto-oxidation processes [66], with the solar irradiance being greater in summer.

### 4.2. Application of the Proposed Fuzzy Methodology to the Seasonal Variations in the Odiel River Hydrochemistry

**Figure 3:**
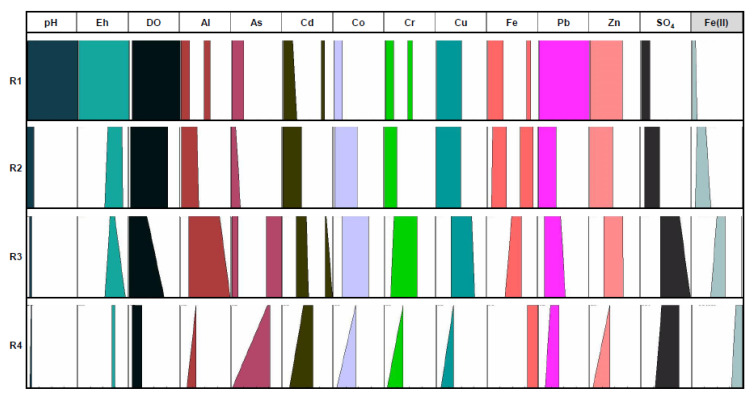
Fuzzy rules related to dry season taking Fe(II) as the consequent.

**Figure 4:**
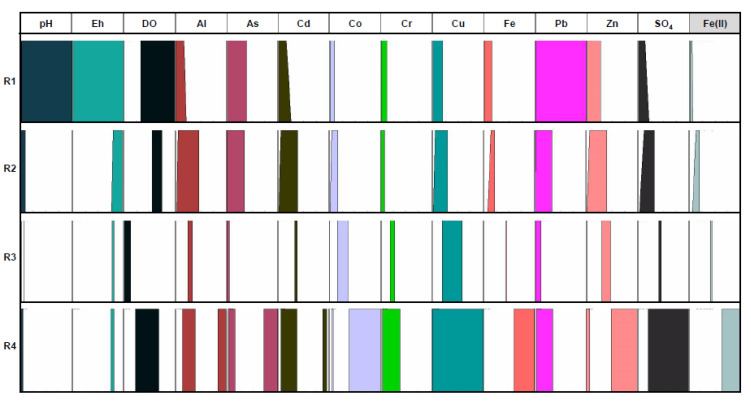
Fuzzy rules related to wet season taking Fe(II) as the consequent.

**Figure 5:**
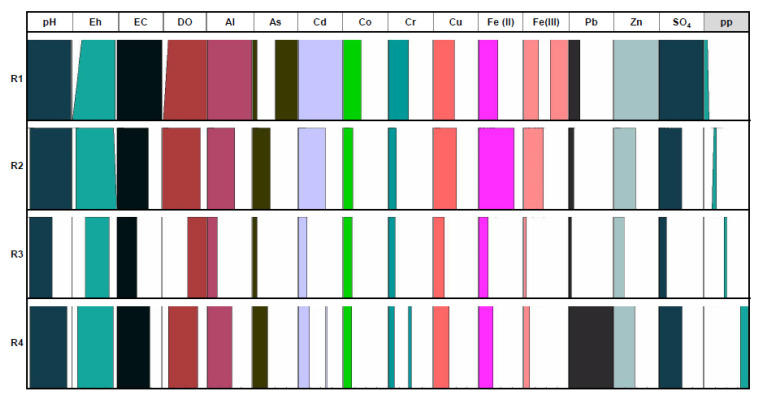
Fuzzy rules taking the accumulated rainfall over 30 days prior to sampling as the consequent.

The graphical fuzzy rules obtained as a result of the selected database processed with the Fuzzy Logic methodology (Figure 3, Figure 4 and Figure 5) have considered the dissolved Fe(II) concentration in the Odiel River Basin in both seasons (dry and wet seasons) and the accumulative rainfall over the 30 days prior to sampling, as consequents parameters (defined by the furthest right columns). This period was established because dumps behave like an aquifer in the sense that they can pour free water into the river for about 20 days. Thus, a period of accumulated rainfall higher than that value (20 days) was set.

The results show how the considered parameters (antecedents) behave in relation to each selected consequent. The four fuzzy rules (R1, R2, R3 and R4) represent the qualitative values that each considered variable can take within its own universe of discourse (Table 2) according to the values assumed by the consequent, demarcated in four ranges (extremely low, low, medium and high). To establish these rules, the algorithm included as eq. (5) was used. These clusters are established in each figure from the values of the parameter taken as a consequent.

Figure 3 displays the graphical fuzzy rules considering as consequent the Fe(II) concentration dissolved in the Odiel River Basin in the dry season. The universe of discourse for Fe(II) values ranges from 0.1 to 1787 mg/L (Table 2). The second rule (R2) could be considered as a special case of the first rule (R1), which refers to two types of waters with very different hydrochemical characteristics, although both have a very low Fe(II) content.

(a) Firstly, those samples that have the highest pH values, low redox potentials and high dissolved oxygen content and also have extremely low contents of Al, As, Cd and other dissolved elements. These samples are from AMD affected waters, that have suffered strong dilution processes, increasing the pH above 5 and decreasing the metallic content by precipitation processes. In this type of water, the metallic content is extremely low and the low value of dissolved Fe will be Fe(II). These waters may contain low concentrations of As in the form of its most reduced species, due to the mobility of this species in more neutral environments [62].

(b) Secondly, samples showing the highest redox potential values and strongly oxic and acidic waters and saturated with dissolved O_2_. These streams are strongly affected by AMD but are not so close to the mining sites. Fe(II) is oxidized to Fe(III) due to bacterial activity and aeration processes when lixiviates flow along surface streams, and therefore, the ratio Fe(II)/Fe decreases; however, the Fe concentration is high as the most oxidized Fe (III) species.

Significant concentrations of dissolved As, Cr and Pb may be associated with the Fe(III) species, due to the high adsorption capacity of these elements on oxidized Fe species [63]. Metal concentrations are not elevated, which is due, in part, to the precipitation of Fe(III)-containing species. In addition, evaporitic sulfate salts are formed during the dry season along the river margins, a process commonly observed in AMD systems [67]. Sulfate concentration is moderate because of the formation of evaporitic sulfate salts, and this involves an increase in the Fe/SO_4_^2−^ ratio for highly polluted samples during the dry season [9].

The third rule (R3) could refer to those streams affected by acid lixiviate that have undergone Fe(III) precipitation processes, which implies a decrease in pH values and an increase in redox potential. These waters may have a high metallic content that will depend on the evaporation processes that occur in the dry season. For this reason, this rule includes the highest ranges of almost all of the elements in the universe of discourse (Table 2) of medium–high values. Elements such as Al, As, Pb, etc., can be found in high concentrations because they are elements that are incorporated into leachates due to acid hydrolysis of the embedding rock. Arsenic and cadmium are associated with polymetallic sulfides and are strongly adsorbed to Fe-oxy-hydroxides; therefore, in this type of water, they could be present in very low concentrations, if the precipitates of Fe oxy-hydroxides are abundant, or in very high concentrations, due to these elements’ desorption processes at very low pH.

Finally, rule R4 refers to samples showing medium–high values of redox potential and low dissolved O_2_ saturation. These streams are strongly affected by the oxidation of sulfides and they are close to the AMD sources, essentially acidic lixiviates that have not undergone dilution or precipitation processes. They are extremely acidic, and the dissolved Fe is mainly Fe (II). All of them show similar characteristics in connection with the elements associated with sulfides’ oxidation, carrying medium–high metals and sulfates concentrations.

Figure 4 displays the graphical fuzzy rules considering dissolved Fe(II) as consequent in the wet season. The universe of discourse for Fe(II) ranges from 0.1 to 1300 mg/L (Table 2). It can be observed that, in the rainy season, the concentration of dissolved elements is governed fundamentally by dilution processes. In addition, during the wet season, the minimum pH value is higher than that in the dry season, and the oversaturation in Fe oxy-hydroxy-sulfates will be also higher [4,65]. Therefore, acid lixiviate with a higher dissolved Fe content will also have a higher content of the other elements and vice versa.

The physicochemical characteristics of the Odiel River Basin undergo significant seasonal changes due to climatological variations [9]. The chemical compositions of these streams also undergo seasonal variations, as described above. Figure 5 shows the graphical fuzzy rules considering the accumulated rainfall as consequent (pp. in the right column). For this, the accumulated rainfall (measured at 23 rain gauges) for the 30-day period prior to each sampling was used to evaluate the seasonal variation of the contamination. In Figure 5, the universe of discourse for accumulated rainfall has a range of 0 to 203 mm (Table 2).

The highest element concentrations occurred in the dry season (R1), which is mainly due to the effects of dissolved salts saturation. As precipitations increased, the dissolved element concentrations (R2) and, consequently the electrical conductivity decreased. It is due to both the dilution effects and to the Fe precipitation and co-precipitation and/or adsorption of other elements on Fe oxy-hydroxy-sulfates. The precipitation of Fe as oxyhydroxides, in turn, causes a decrease in pH (R3). Furthermore, the electrical conductivity and concentration of dissolved elements increase when the precipitation is more abundant (R4), coinciding after the first autumn rainfall [10,53], due to the redissolution and rinse out of soluble efflorescent sulfate salts precipitated during the dry season along the river margins.

## 5. Conclusions

In this work, the Fuzzy Logic technique was used to analyze the seasonal evolution in the Odiel River Basin, strongly affected by AMD pollution. With the use of Fuzzy Logic tools, qualitative models were presented that allowed for hydrochemical characterization of the Odiel River Basin affected by AMD. In addition, this has allowed a more grounded analysis of the seasonal influence of the rainfall regime on these processes.

To carry out this analysis, Fe(II) and accumulated rainfall over 30 days were taken as a consequence, which made it possible to evaluate these two important parameters that influence the seasonal evolution of the Odiel River Basin. Based on this study, the following conclusions have been obtained:

Metal concentrations are affected by dilution processes in the wet season, having been found in much higher concentrations than most of the metals in the dry season (in maximum and average values), except for arsenic and lead. Thereby, an increase in Pb was observed in the wet months, and a decrease in As concentrations was observed in summer. Moreover, the minimum pH values were higher in the wet season than in the dry season.

Coinciding with the period of higher rainfall (in this area, this happens with the first rains in the beginning of autumn, in the wet season), an increase in EC and metal concentrations was confirmed, due to dissolution of evaporitic salts of the riverbank.

Due to the fact that the analysis by Fuzzy Logic allows for evaluating the relationships of different parameters with each other, and, fundamentally, with the parameter taken as a consequent, it was possible to determine that, while the lixiviates with a higher Fe content have higher metal concentrations in the wet season (especially Zn, Cu, Co, Cd and Al), in the dry season, this situation is inversed.

The hydrochemical characteristics are greatly affected by seasonal changes, with a higher pH in the wet season (up to 8.59) and with higher metal concentrations in the dry season (up to 4000 mg/L of Fe (II)). This coincides with what was found by other authors for other rivers of the Iberian Pyrite Belt, such as the Cobica River and the Tinto River.

The conclusions obtained in this work are in agreement with those obtained by Sarmiento et al. [9], showing that the hydrochemical characteristics in the Odiel River Basin are closely related to the concentrations of dissolved Fe species, as well as to the rainfall regime. On the other hand, the use of the Fuzzy Logic tool is clearly validated to obtain conclusions with hydrochemical data in extreme environments, such as those contaminated by acid mine drainage.

## Figures and Tables

**Table 1:** Statistical summary of the parameters analyzed at 37 sampling points in the Odiel River Basin.

Parameters	Annual Range	Wet Season (*n* = 111)				Dry Season (*n* = 121)			
		**Min**	**Max**	**Mean**	**SD**	**Min**	**Max**	**Mean**	**SD**
pH	2.12–8.77	2.49	8.59	4.14	1.48	2.12	8.77	3.79	1.62
Eh (mV)	211–813	211	781	594	130	259	813	621	129
EC (mS/cm)	0.1–18.5	0.1	13.7	2.0	2.79	0.2	18.5	3.5	4.05
DO (%)	26–122	28	122	90	13.9	26	122	87	17.8
Al (mg/L)	bdl–2045	bdl	1139	82	197	bdl	2045	186	347
As (µg/L)	bdl–7466	bdl	7466	245	889	bdl	3817	162	555
Cd (µg/L)	bdl–2249	bdl	1446	107	255	bdl	2249	207	383
Co (µg/L)	bdl–30,869	bdl	15,761	782	2210	bdl	30,869	1646	3800
Cr (µg/L)	bdl–926	bdl	477	27	78	bdl	926	41	103
Cu (mg/L)	bdl–321	bdl	192	12	30	bdl	321	22	43
Fe (mg/L)	bdl–4282	bdl	2003	133	326	bdl	4282	317	690
Fe(II) (mg/L)	bdl–4000	bdl	1756	107	287	bdl	4000	213	567
Mn (mg/L)	bdl–374	bdl	220	16.8	37.6	bdl	374	38.4	65.5
Mo (µg/L)	bdl–467	bdl	240	14	35	bdl	467	44	81
Ni (µg/L)	bdl–14,429	bdl	6839	413	1104	bdl	14,429	937	2080
Pb (µg/L)	bdl–5930	bdl	5930	275	732	bdl	1501	178	257
Sb (µg/L)	bdl–1041	bdl	623	30	93	bdl	1041	82	154
Sn (µg/L)	bdl–496	bdl	46	3	6.2	bdl	496	45	81.8
Zn (mg/L)	bdl–860	bdl	402	31	74	bdl	860	70	134
SO_4_ (mg/L)	10–36,397	11	19,332	1629	3600	10	36,397	3729	6227

Eh, redox potential; EC, electrical conductivity; DO, dissolved oxygen; Min, minimum; Max, maximum; SD, standard deviation; bdl, below detection limit. Note: the values below detection were replaced by the bdl value itself.

**Table 2:** Universe of discourse—range of parameter values—for data mining.

Parameters	Wet Season	Dry Season	Total
pH	2.5–8.6	2.1–8.8	2.1–8.8
Eh (mV)	211–781	259–813	211–813
EC (µS/cm)			115–18,480
DO (%)	28–122	40–122	26–122
Al (mg/L)	0.1–1139	0.1–1614	0.1–1045
As (µg/L)	2–3487	2–2006	2–7466
Cd (µg/L)	2–1446	2–1605	2–2249
Co (µg/L)	2–15,761	2–14,478	2–30,869
Cr (µg/L)	2–477	2–446	2–926
Cu (mg/L)	0.1–192	0.1–164	0.1–321
Fe (mg/L)	0.1–1528	0.1–2085	
Fe (II) (mg/L)	0.1–1300	0.1–1787	0.1–4000
Fe (III) (mg/L)			0.1–1757
Pb (µg/L)	2–5930	2–1501	2–5930
Zn (mg/L)	0.1–402	0.1–584	0.1–860
SO4 (mg/L)	11.1–19,332	9.9–24,155	9.9–36,397
pp30 (mm) *			0–203

* pp 30 means the accumulative rainfall over 30 days prior to sampling.

## Data Availability

Publicly available datasets were analyzed in this study. This data can be found here: University of Huelva repository, http://hdl.handle.net/10272/7629.
